# 3^rd^ Etnean Occupational Medicine Workshop—Breast Cancer and Work

**DOI:** 10.3390/cancers12071925

**Published:** 2020-07-16

**Authors:** Venerando Rapisarda, Caterina Ledda

**Affiliations:** Occupational Medicine, Department of Clinical and Experimental Medicine, University of Catania, 95124 Catania, Italy; vrapisarda@unict.it

Breast cancer, the most frequently occurring cancer in women, is a major public health problem, with over 1 million estimated new cases worldwide and nearly 459,000 related deaths every year [1]. Breast cancer is highly heterogeneous in its pathological characteristics, with some instances showing slow growth with an excellent prognosis, while others involve aggressive tumors with widespread metastasis. Current predictions and statistics suggest that both the worldwide incidence of breast cancer and related mortality are on the rise. It has been predicted that the global incidence of female breast cancer will reach approximately 3.2 million new cases per year by 2050 [1]. These numbers reflect the magnitude of breast cancer incidence, its effect on society worldwide, and the urgent need for preventive and treatment measures. Breast cancer incidence and death rates generally increase with age; most of them are diagnosed in women over 50 [1].

In particular, ductal carcinoma in situ shows overlapping epidemiology with invasive ductal carcinoma of the breast, sharing similar risk factors such as age, mammographic density, family history and hormonal therapy, as well as genetic factors such as BRCA1/BRCA2, histotypes and molecular subtypes, such as luminal A and B, HER2-enriched, and basal-type [2]

*BRCA1* and *BRCA2* genes are involved in DNA double-strand break repair and relate to breast cancer. Shift work is associated with biological clock alterations and with a higher risk of breast cancer. Bracci et al. [3] investigated the variability of expressions of *BRCA* genes throughout the day in healthy subjects, and measured *BRCA* expression levels in shift workers. Moreover, they measured the expression of these genes in lymphocytes from a group of shift and daytime workers. The change in 24-hour expression levels of *BRCA1* and *BRCA2* genes was statistically significant, decreasing from the peak at midday to the lowest level at midnight. Lower levels for both genes were found in shift workers compared to daytime workers. The diurnal variability of *BRCA1* and *BRCA2* expression suggests the relation of the DNA double-strand break repair system with the biological clock. The lower levels of *BRCA1* and *BRCA2* found in shift workers may be one of the potential factors related to a higher risk of breast cancer [3].

However, some breast cancer risk factors may be preventable. Traditional risk factors for breast cancer include reproductive status, genetic mutations, family history and lifestyle. However, increasing evidence has identified an association between breast cancer and occupational/environmental factors.

Breast cancers include a heterogeneous group of diseases with clinical behaviors that may vary according to the hormonal receptor status. However, limited knowledge is available on the role of breast cancer environmental and occupational risk factors, as regards the onset of specific molecular disease phenotypes. In a systematic review, Leso et al. [4] found that some positive associations were reported between solvent, polycyclic aromatic hydrocarbon, organophosphorus insecticide and synthetic fiber exposure and estrogen receptor-positive cases, while other investigations either demonstrated a relationship with receptor-negative tumors, or failed to detect any significant effects. 

Furthermore, in occupational medicine, a significant advance in medical practice is the incorporation of the Clinical Decision Support Systems to assist and support healthcare staff in clinical decision-making, thus improving the quality of decisions and overall patient care while minimizing costs [5]. 

Occupational physicians, therefore, need to face the return to work and the fitness for work of workers previously diagnosed with breast cancer with a sufficient cultural and technical background. In addition to individual characteristics preceding the diagnosis, clinical outcome, lifestyles and occupational variables are the most impactful factors on return to work, which need to be considered [6]. Scientific evidence suggests that a multidisciplinary approach would be preferred. Occupational health professionals should take note of individual and collective risk assessments, promote a healthy lifestyle (i.e., Mediterranean diet) before and after sick leave, encourage rehabilitation, and propose solutions to improve the interactions between employees and the workplace. Encouraging patients with low-stage diagnoses to return to work could be useful, enhancing their quality of life, and reducing days of sick leave and requests for disability pension. High-stage patients could need more time to recover from chemotherapy; however, they should be encouraged if their general condition allows for it [6,7].
